# Chronic Rejection and Extrahepatic Biliary Tract Obstruction 8 Years After Orthotopic Liver Transplantation Using the Gallbladder-Conduit Technique

**DOI:** 10.1155/1991/65497

**Published:** 1991

**Authors:** J. Lerut, A. Zimmermann, Ph. Gertsch, R. Preisig, L. H. Blumgart

**Affiliations:** 1 Department of Visceral and Transplantation Surgery Institute of Pathology University Hospital Inselspital Berne CH-3010 Switzerland; 2 Department of Clinical Pharmacology University Hospital Inselspital Berne CH-3010 Switzerland; 3 Department Surgery Liver transplantation- University Hospital St Luc Cath. Univ. Louvain UCL. Av. Hippocrate 10 BRUSSELS 1200 B Belgium

## Abstract

A case of delayed biliary obstruction and cholangitis, occurring in the setting of chronic allograft rejection, 8 years after liver transplantation using the gallbladder-conduit, is presented. Extrahepatic biliary obstruction may be seen in the late follow-up of liver grafting and rejection phenomena may play a significant role in the development of such obstruction.


HPB Surgery, 1991, Vol. 5, pp. 17–22
Reprints available directly from the publisher
Photocopying permitted by license only

© 1991 Harwood Academic Publishers GmbH
Printed in the United Kingdom

### CHRONIC REJECTION AND EXTRAHEPATIC BILIARY TRACT OBSTRUCTION 8 YEARS AFTER ORTHOTOPIC LIVER TRANSPLANTATION USING THE GALLBLADDER-CONDUIT TECHNIQUE

J. LERUT, A. ZIMMERMANN*, Ph. GERTSCH, R. PREISIG** and L.H. BLUMGART

*Department of Visceral and Transplantation Surgery, Institute of Pathology*, Department of Clinical Pharmacology**, University Hospital, Inselspital, CH - 3010, Berne*

*(Received 11 October 1990)*

A case of delayed biliary obstruction and cholangitis, occurring in the setting of chronic allograft rejection, 8 years after liver transplantation using the gallbladder-conduit, is presented. Extrahepatic biliary obstruction may be seen in the late follow-up of liver grafting and rejection phenomena may play a significant role in the development of such obstruction.

KEY WORDS: Liver transplantation, chronic rejection, biliary complication, gallbladder conduit, primary biliary cirrhosis, recurrent disease

### INTRODUCTION

Following the early use of orthotopic liver transplantation (OLT) 30 to 50% of patients developed severe complications related to the biliary tract reconstruction[1,2,3]. In order to reduce the incidence of biliary complications, Waddell and Calne, developed the concept of the gallbladder-conduit between donor bile duct and recipient bile duct or jejunum[4,5]. A case of delayed biliary obstruction and cholangitis, associated with a stricture involving the gallbladder-conduit, occurring with chronic allograft rejection 8 years after liver transplantation, is presented.

### CASE REPORT

A 36-year-old woman underwent OLT for end-stage primary biliary cirrhosis (PBC) on December 12, 1980, in Cambridge (R.Y. Calne). The biliary tract reconstruction was with a gallbladder-conduit and T-tube. Post-transplant T-tube-cholangiography was normal and the biliary stent was removed after four months. Immunosuppression consisted of cyclosporine and low-dose steroids. In 1983 a biopsy-proven episode of allograft rejection was managed by increasing the

Address correspondence to: PD Dr. Jan Lerut, Department Surgery, Liver transplantation – University Hospital St Luc – Cath. Univ. Louvain UCL. Av. Hippocrate 10 BRUSSELS – 1200 B-Belgium steroid dose and adding azathioprin. Although the titer of antimitochondrial antibodies remained the same as preoperatively (1/100), a recurrence of the primary disease was not demonstrated.

The patient remained very well until the summer of 1988, when she developed fever and jaundice. On clinical examination, the right upper quadrant was tender. Bilirubin had risen to 114 mM/l, alakaline phosphatase to 916 IU/l, GGT to 3393 IU/l, SGOT and SGPT to 145 and 222 IU/l.

HIDA-scan showed a reduced uptake of the tracer by the allograft and a marked delay of clearance from the biliary system. Duplex-ultrasonography did not detect any parenchymal, biliary or vascular abnormalities.

Because of a further bout of cholangitis two months later, the patient was rehospitalized. Repeated blood cultures were sterile. Endoscopic retrograde cholangiography (ERC) showed a normal distal bile duct and donor gallbladder, with a patent, prominent, cystic duct and a stricture at the anastomosis between donor common bile duct and Hartmann's pouch of the donor gallbladder. The extrahepatic biliary system, proximal to the stenosis, was slightly dilated; the intrahepatic biliary tree did not show any signs of chronic rejection (Figure 1).

Percutaneous allograft liver biopsy showed typical signs of chronic rejection.

A cholestatic syndrome with cholangitis due both to partial biliary obstruction and chronic allograft rejection was diagnosed. Because the patient refused retransplantation at that time, it was decided to perform a surgical correction of the biliary obstruction in order to improve the patient's condition and to avoid further infection. After opening the gallbladder it could be seen that the proximal choledocho-cholecystostomy was almost completely closed. Removal of the distal part of the donor bile duct and of the donor gallbladder created a situation in which a normal donor proximal bile duct and a normal recipient distal bile duct were widely separated from each other. An isolated transmesocolic jejunal segment of 8 cm length was interposed in an isoperistaltic fashion between the separated ductal orifices.

Liver biopsy confirmed the chronic allograft rejection expressed by typical vanishing bile duct syndrome. Bacteriological examination of bile was sterile.

*Pathological examination of the donor gallbladder and of the distal donor bile duct*: The mucosal layer of the gallbladder was well preserved but slightly atrophic. The main finding consisted of an arteriopathy, characterized by intimal fibrosis with, in places significant stenosis (Figures 2A and B).

One arterial branch showed foam cells within the fibrotic intima, and another arterial segment exhibited lymphocytic infiltrations (Figure 2A). Lymphocytes and a few plasma cells were also clustered around small vessels both within the mucosa and the muscle layer. In addition, the gallbladder wall showed diffuse fibrosis. The specimen representing the resected afferent branch with the biliary stricture revealed arterial changes as well, with intimal fibrosis and accumulation of foam cells (Figure 2C). Biliary glands were distorted, and there were focal lymphocytic infiltrates (Figure 2D). These changes were interpreted as representing chronic rejection with graft arteriopathy.

HIDA-scan, performed three months after operation, confirmed the poor uptake of the tracer by the allograft but a rapid activity of the tracer in the small intestine. There was no evidence of biliary tract obstruction. Ten months after correction of the biliary stricture, the patient remained well and afebrile; the signs of chronic rejection however persisted.

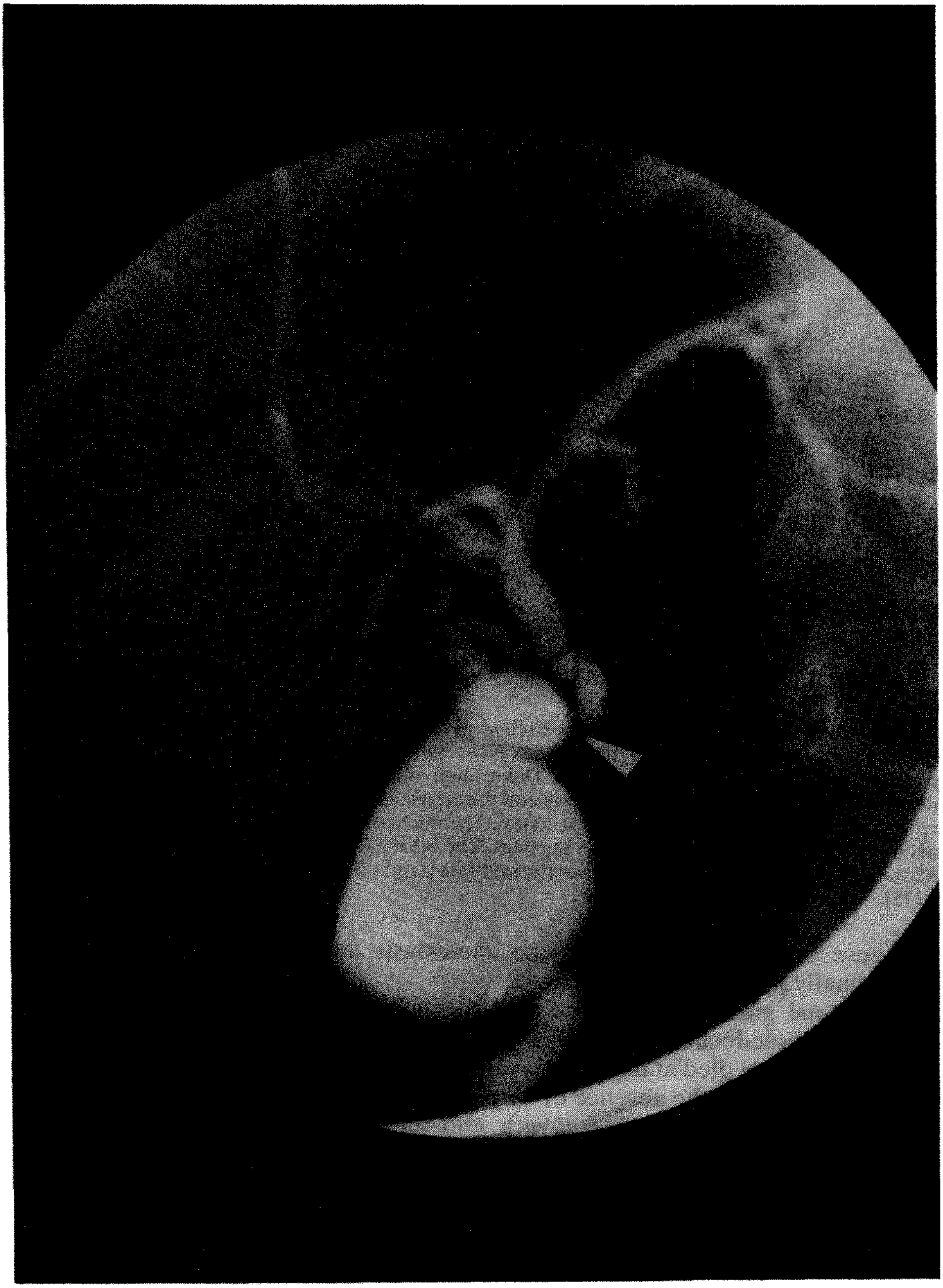

**Figure 1** Preoperative ERC shows a tight stricture at the proximal anastomosis of the gallbladder-conduit compensated by a dilated cystic duct.

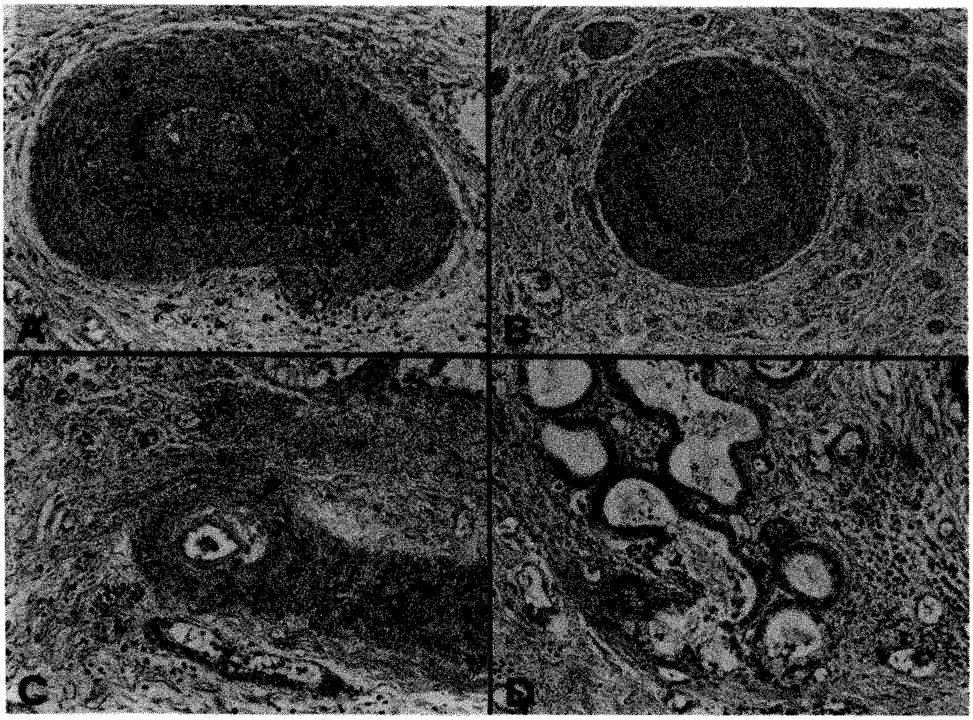

**Figure 2** Pathological examination of the excised gallbladder-conduit and the distal part of the donor bile duct. A: Medium-sized gallbladder artery. Note the fibrous thickening of the intima and lymphocytes infiltrating one sector of the vessel. B: This arterial branch shows severe fibrosis of the intima, with luminal stenosis. The internal elastic lamina and the media are preserved. C: Small artery in a bile duct segment resected from the site of biliary stricture. The thickened intima shows an accumulation of foam cells (arrowhead). D: Resected bile duct segment. The biliary glands are distorted and contain exudate. The connective tissue shows focal lymphocytic infiltrates (right half of figure). (H and E stain, all figures × 175).

Because of the progression of the cholestasis and general deterioration, the patient finally underwent regrafting.

The jejunal graft was removed and the biliary reconstruction consisted of a choledocho-choledochostomy with T-Tube stent. After a good initial postoperative course, the patient died at day 25 because of an acute extensive subcapsular hepatic necrosis occurring in the absence of vascular thrombosis. Pathological examination of the first graft was consistent with end-stage liver rejection with organized thrombosis of intra- and extra hepatic arteries. There were no signs of recurrent PBC. The vascular lesions of the liver corresponded with those found some months earlier in the gallbladder-conduit and adherent bile duct.

### DISCUSSION

Because of the high incidence of biliary complications after OLT, a technique of biliary tract reconstruction was introduced in the mid-seventies in which the gallbladder was interposed between the donor and recipient bile ducts[4,5].

This technique allowed the reconstruction of a tension-free biliary anastomosis, preservation of the sphincter of Oddi as well as the formation of a large anastomosis. This method is now only regularly used in the King's College-Cambridge liver transplant program with a reported complication rate of 12%[6,7]. Most complications occur during the early postoperative period and biliary strictures are reported in 7 of 196 patients (4%). All strictures occurred at the anastomosis between the donor common bile duct and Hartmann's pouch. An area of relative ischemia at this anastomosis has now been discussed as an etiological mechanism[7]; however in 3 of the 7 reported patients the obstruction occurred in the setting of chronic allograft rejection. Thus, rejection phenomena in the biliary tract may play a significant role as a factor in late complications and should be searched for in patients with delayed appearance of functional liver disturbance.

The case reported shows that extrahepatic biliary obstruction may be due to chronic rejection but also demonstrates that allograft rejection and biliary complications may occur together at late follow-up.[8,9,10,11]. Chronic rejection of the allograft may be prolonged so that it may be useful in the setting of chronic rejection associated with biliary obstruction, to relieve the cholestatic syndrome in order to avoid septic complications in an immunosuppressed patient.

It should be mentioned that pathological examination of the first graft was compatible with irreversible graft rejection although this patient has been included recently in a group of patients presenting evidence of recurrent disease after OLT for PBC[12].

The use of standard techniques of biliary reconstruction has considerably reduced the incidence of biliary complications in OLT[6,13,14,15,16,17].

Choledocho-choledochostomy with a T-tube stent or choledocho-jejunostomy Roux-en-Y are the preferred methods of biliary tract reconstruction. The gallbladder-conduit is reserved for individual cases in which adequate mobilization of a Roux-Y-jejunal loop to the donor bile duct is difficult or impossible because of previous abdominal operations.

### *References*

1. Starzl, T.E., Ishikawa, M. and Putnam, C.W., *et al.* (1974) Progress and deterrents to orthotopic liver transplantation, with special reference to survival, resistance to hyperacute rejection and biliary duct reconstruction. *Transplant. Proc.*, **6**, 129–139
2. Starzl, T.E., Putnam, C.W. and Hansbrough, J.F., *et al.* (1977) Biliary complications after liver transplantation, with special reference to the biliary cast syndrome and techniques of secondary duct repair. *Surgery*, **81**, 212–221
3. Starzl, T.E., Iwatsuki, S. and Van Thiel D.H., *et al.* (1982) Evolution of liver transplantation. *Hepatology*, **2**, 614--636
4. Waddell, W.R. and Grover, F.L. (1973) The gallbladder as a conduit between the liver and intestine. *Surgery*, **74**, 524–529
5. Calne, R.Y. (1976) A new technique for biliary drainage in orthotopic liver transplantation utilizing the gallbladder as a pedicle graft conduit between donor and recipient common bile ducts. *Ann. Surg.*, **184**, 605–609
6. Rolles, K. (1987) Biliary tract complications. In: Liver Transplantation (second edition), R.Y. Calne ed. (pp.473–483) New York: Grune and Stratton
7. Rolles, K. (1983) Early biliary tract complications. In: Liver Transplantation, (first edition), R.Y. Calne (Ed)., (pp.319–326) New York: Grune and Stratton
8. Scudamore, C.H. (1983) Late biliary tract complications. In: *Liver Transplantation* (first edition), R.Y. Calne (Ed)., (pp.327–341) New York: Grune and Stratton
9. Lerut, J., Demetris, A.J. and Stieber, A.C., *et al.* (1988) Intrahepatic bile duct strictures after human orthotopic liver transplantation. *Transplant. Int.*, **1**, 127–130

10. Wraight, E.R. (1987) Radionuclide imaging in postoperative assessment. In: *Liver Transplantation* (second edition), R.Y. Calne (Ed)., (pp.293–299) New York: Grune and Stratton
11. Zajko, A.B., Campbell, W.L. and Bron, K.M., *et al.* (1989) Diagnostic and interventional radiography in liver transplantation. *Gastroent. Clin. N. Am.*, **17**, 105–143
12. Polson, R.J., Portmann, B. and Neuberger, J., *et al.* (1989) Evidence of disease recurrence after liver transplantation for Primary Biliary cirrhosis. *Gastroenterology,* **97**, 715–725
13. Bismuth, H., Castaing D. and Gugenheim, J., *et al.* (1987) Roux-en-Y hepatojejunostomy: A safe procedure for biliary anastomosis in liver transplantation. *Transplant. Proc.,* **19**, 2413–2415
14. Lerut, J., Gordon, R.D. and Iwatsuki, S., *et al.* (1987) Biliary tract complications in human orthotopic liver transplantation. *Transplantation,* **43**, 47–51
15. Krom, R.A.F., Kingma, L.M. and Haagsma, E.B., *et al.* (1985) Choledocholedochostomy, a relatively safe procedure in orthotopic liver transplantation. *Surgery,* **97**, 552–556
16. Ringe, B., Oldhafer, K. and Bunzendahl, H., *et al.* (1989) Analysis of biliary complications following orthotopic liver transplantation. *Transplant. Proc.*, **21**, 2472–2476
17. Wolff, H., Otto, G. and David H. (1985) Biliary tract reconstruction in liver transplantation. *Transplant. Proc.,* **17**, 274–275

*(Accepted by S. Bengmark 11 October 1990)*

